# Exploring the Factors that Influence Workforce Participation for People with Multiple Sclerosis: A Discrete Choice Experiment

**DOI:** 10.1007/s10926-020-09952-5

**Published:** 2021-01-27

**Authors:** Elizabeth Goodwin, Annie Hawton, Jennifer A. Whitty, Colin Green

**Affiliations:** 1 Health Economics Group, Institute of Health Research, University of Exeter Medical School, University of Exeter, Exeter, UK; 2 NIHR Applied Research Collaboration-South West (PenARC), University of Exeter Medical School, University of Exeter, Exeter, UK; 3 NIHR Research Design Service South West, University of Exeter Medical School, University of Exeter, Exeter, UK; 4 Health Economics Group, Norwich Medical School, University of East Anglia, Norwich, Norfolk UK; 5 NIHR Applied Research Collaboration (ARC), East of England, Cambridge, UK

**Keywords:** Multiple sclerosis, Employment, Surveys and questionnaires

## Abstract

*Purpose* Research indicates that employment is beneficial for people with multiple sclerosis (MS). However, people with MS typically face reduced workforce participation compared to the general population. Using a discrete choice experiment (DCE) we explored which factors are most important in influencing employment choices of people with MS, and whether the relative importance of factors differs between subgroups. *Methods* Attributes and levels for the DCE were developed using a systematic literature review and public involvement techniques with people with MS. In an online survey, respondents were asked to choose between two hypothetical job scenarios described using six attributes. We used a large, national register (the UK MS Register), to recruit participants aged 18–64 years with a diagnosis of MS. Choice data were analysed using multinomial logit and latent class models. *Results* Analyses were based on responses from 2350 people with MS. The preferred model specification was a latent class model, with three classes of respondent. The relative importance of attributes varied between classes, with one giving the greatest weight to the impact of work on other aspects of their lives, the second to having supportive bosses and colleagues, and the third to job flexibility. The classes differed significantly in terms of age and gender, type of MS, and socio-economic status. *Conclusions* Significant heterogeneity was apparent among people with MS regarding the factors that influence their employment decisions. Attributes concerning the impact of work, attitudes in the workplace and job flexibility appear more influential than those concerning physical workplace adaptations.

**Supplementary Information:**

The online version of this article (10.1007/s10926-020-09952-5) contains supplementary material, which is available to authorized users.

## Introduction

Multiple sclerosis (MS) is the most common cause of neurological disability within the young adult population of the UK [1]. In the majority of cases, the disease course is initially characterised by periods of relapse and remission (relapsing-remitting MS), eventually becoming progressive (secondary progressive MS), while others experience a progressive disease course from the outset (primary progressive MS) [2]. MS causes a wide range of symptoms (including fatigue, mobility restrictions, cognitive problems and mood disorders), which vary considerably between individuals, and is associated with decrements across all domains of health-related quality of life [3].

In addition to providing financial security, employment can contribute significantly to the physical and mental wellbeing of people with MS (PwMS), providing a source of support and social interaction and a sense of identity and purpose [4]. The onset of MS typically occurs between the ages of 20–40 [5], with the majority of people in employment at time of diagnosis. However, many PwMS face challenging work lives, reporting higher rates of unemployment and part-time employment, reduced labour force participation and lower incomes, compared to the general population and people with other long-term physical health conditions [6]. A recent report by the UK All-Party Parliamentary Group (APPG) on MS suggests that as many as 80% of PwMS retire within 15 years of diagnosis, gradually reducing or adapting their work over time before leaving the workforce permanently [7].

This study aims to use a choice-based method to understand which factors are most important in influencing employment choices of PwMS, with a particular focus on workplace-related issues. Given the variation in how MS affects individuals [3], we also aim to explore how the relative importance of factors may differ between subgroups of PwMS. This is with a view to informing employment policies and good practice.

## Background

The research reported here is informed by a systematic review of studies using qualitative methods to explore employment participation by PwMS (see Online Resource 1). These studies identified several factors that influence the choices of PwMS regarding employment, outlined below.

A number of specific symptoms associated with MS have been found to influence participation in employment, particularly fatigue, cognitive issues, mobility limitations, bladder or bowel dysfunction and visual problems [8–12]. However, it is the *effect* of symptoms, rather than the symptoms per se, that are most relevant, including effects on overall productivity, daily work capacity, and specific job duties [8, 9, 11].

In terms of the working environment, a key aspect is workplace culture, specifically having a supportive employer and understanding colleagues, as opposed to experiencing discrimination, harassment or work-related stress [8–10, 13, 14]. Another is the availability of practical support, including the provision of adaptations and assistive technology, job flexibility and accommodations. Also relevant are the ease of travelling to work and physically accessing the workplace [9–15].

Personal and emotional factors are also important. Having a supportive home environment can be crucial in helping PwMS to balance work and non-work activities and responsibilities [9, 10, 13]. Some PwMS have reported the emotional effects of working less or not at all, of feeling they are no longer doing their job well, or of feeling like a burden to their co-workers [8, 16].

Other factors relate to the cultural and societal environment, including disability benefits, appropriate employment, social and health services, and societal expectations regarding the employment of people with long-term health conditions [8, 11, 13, 14].

Overall, previous research has identified various factors that influence the employment decisions of PwMS [11]. A key challenge for PwMS is to balance the benefits of working with the costs, in terms of potential exacerbation of symptoms or negative impacts on other aspects of life [10].

## Methods

### Discrete Choice Experiment

We use a discrete choice experiment (DCE) to explore which factors are most important in influencing the employment choices of PwMS. The DCE framework assumes that products (or any options that people can choose between) can be defined according to a set of characteristics, or “attributes”; such that the demand for a product comprises a demand for a particular combination of attributes [17]. Each attribute has a fixed number of levels, and each combination of levels across all attributes describes a unique product or scenario. In a DCE, respondents are asked to choose between two or more options, described in terms of their key attributes [18]. Analysing this type of stated preference data allows the relative importance of the attributes to be estimated [19]. DCEs have been applied to human resources policy questions, particularly in investigating which aspects of a job are most influential in guiding a (potential) worker’s decision whether or not to take up a particular post [20].

### Attributes

**Fig. 1:**
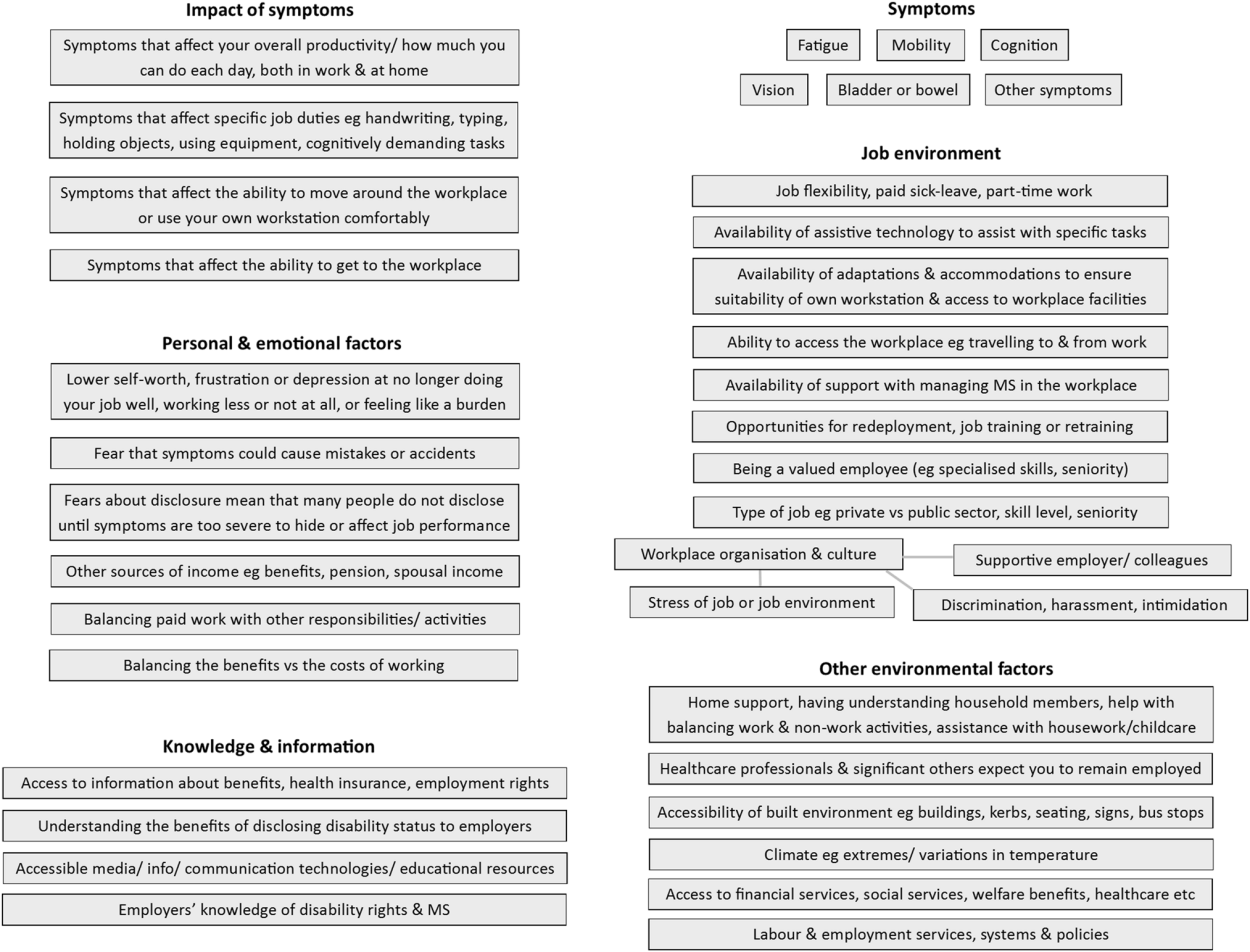
Conceptual framework of factors that influence the employment decisions of people with multiple sclerosis

Best practice principles recommend that the development of DCE attributes should be informed by the findings of qualitative research, undertaken with the target population for the study [21]. We undertook a scoping search for published qualitative and quantitative studies, which indicated that a considerable amount of qualitative research had been undertaken in this area, and that the qualitative studies provided a more comprehensive and relevant basis for attribute development, compared to the quantitative studies. Therefore, we undertook a full systematic review of existing qualitative research (outlined above) and used this to develop a conceptual framework of factors that have been found to influence workforce participation by PwMS. This framework (Fig. 1) formed the basis for an interactive exercise with a group of PwMS (n = 6), from an established Patient Involvement Group.

Each individual was provided with a copy of the conceptual framework, consisting of the categories and factors printed onto separate cards and arranged on a magnetic whiteboard. Individuals were asked to identify any omissions, duplications or links between factors, before using their cards and whiteboards to arrange the factors in order of importance to them. After the individual exercises, group discussions were held to produce a joint list and to organise the selected factors into categories, aiming to generate attributes that were (1) suitable for inclusion in a DCE, (2) considered to be of high importance by PwMS, and (3) as far as possible, amenable to change by employers or other organisations.

Six attributes were selected to describe employment scenarios in the DCE: Flexibility (two levels), Alterations (three levels), Culture (two levels), Travel (two levels), Impact (two levels) and Salary (three levels). The number, range and descriptions of the levels were generated and refined through an iterative process with the Group. More information on attribute development is provided in Online Resource 1. The attributes and levels for the DCE, and the definitions presented to respondents, were as follows.

Flexibility of job (*flexibility*). This factor relates to adjustments that can be made to the job, to adapt to your needs. These can include working part-time or flexible hours, or changing the amount and type of work you are expected to do.

1. The job can be adjusted to accommodate your needs as they change or fluctuate.
2. The job cannot be adjusted to accommodate your needs as they change or fluctuate.

Alterations in the workplace (*alterations*). This factor relates to alterations that can be made in the workplace. These can include help with getting around at work, making sure you can access facilities (e.g. toilets), and providing specialist equipment.

1. Alterations are made that completely meet your current needs.
2. Some alterations are made, but they don’t completely meet your needs.
3. No alterations are made.

Workplace culture (*culture*). This factor relates to attitudes and behaviour in the workplace. This includes how supportive your employer and colleagues are, and how well they understand about how your MS affects you.

1. Your bosses and colleagues understand about your MS and are supportive.
2. Your bosses and colleagues do not understand about your MS and are not supportive.

Getting to work (*travel*). This factor relates to your ability to get to and from the workplace. This includes having access to suitable transport given the physical location of the workplace.

1. It is usually easy to get to the workplace.
2. It is usually difficult to get to the workplace.

Impact of working (*impact*). This factor relates to the impact of your work on other aspects of your life. This could be positive (e.g. financial security, sense of purpose) or negative (e.g. making some of your symptoms worse, not leaving you enough energy to do other things).

1. Overall, work has a positive impact on other aspects of your life.
2. Overall, work has a negative impact on other aspects of your life.

Salary: Take-home pay (after tax deductions).

1. £100 per month more than the average pay for this type of job.
2. The same as the average pay for this type of job.
3. £100 per month less than the average pay for this type of job.

### Experimental Design

The attributes are capable of describing 144 scenarios, and a total of 10,296 unique choice pairs. We used a modified Fedorov algorithm to select a subset of choice pairs, sufficient to estimate main effects and two-factor interactions for one attribute using a linear model [22, 23]. The permitted interactions were between the Impact attribute and each of the other attributes, as these were considered to be the most likely interactions. The experimental design consisted of 72 choice pairs (there was some duplication of individual scenarios across the pairs). A previous choice-based survey undertaken with this population indicated that PwMS are able to respond to six choice sets [24], therefore each respondent answered six DCE questions. This required 12 versions of the survey. Respondents were allocated a version of the survey at random.

### Procedure

Before starting the DCE, participants were given instructions and undertook a practice DCE question. The instructions provided, and an example DCE question, are included in the Appendix. Participants were asked to imagine that they had been offered two jobs, Job A and Job B, which were described using the attributes presented above, and to decide which of the two they would prefer. A forced choice was selected over an opt-out design in this context, due to concerns that respondents who were currently unable to work might always choose to “opt-out”, which would have been problematic given the high proportion of the sample who were not working. Here, the aim was to determine the relative importance of the attributes, rather than to predict how PwMS might behave when faced with employment decisions, therefore the potential advantages of an opt-out design were less relevant. The survey design was informed by an online pilot with 47 members of the UK MS Register.

### Sample Size and Recruitment

The survey was administered online, a mode of administration that is frequently used for DCEs [25]. Respondents were recruited from the UK MS Register, which had over 14,000 members in March 2017 [26], who have been shown to be broadly representative of PwMS in the UK in terms of gender, age and MS type [27, 28]. Members of the Register are requested to provide information via an internet portal on a quarterly basis, including demographic and clinical information as well as a range of patient-reported outcome measures, capturing data on quality-of-life and the incidence and severity of particular symptoms [26]. Information on the socio-demographic and clinical characteristics of respondents was linked anonymously to the survey data by the MS Register. This included age, gender, type of MS (relapsing-remitting, secondary progressive, primary progressive), date of diagnosis with MS, and most recent responses to the Multiple Sclerosis Impact Scale (MSIS-29), a patient-reported measure of health-related quality of life with two subscales representing physical and psychological health [29]. Respondents were asked to provide their current employment status and to categorise their current or most recent job using the National Statistics Socio-Economic Classification (NS-SEC) [30].

Because this survey focuses on employment, invitations were sent to members of usual working age, i.e. 18–64 years. Inclusion was not restricted to those currently in employment, as responses were required from those who had ceased employment early or are temporarily absent from the workforce. Ethical approval was granted by the University of Exeter Medical School Research Ethics Committee.

As a starting point for determining our target sample size, we used a simple random samples strategy, in which each individual in the sampling frame has an equal likelihood of being selected for the sample (31). This approach provides a suggested minimum sample size, estimated using the following formula:

$$\varvec{N} \ge \frac{1-\varvec{p}}{\varvec{T}\varvec{p}\left({\varvec{a}}^{2}\right)} \left[{\varvec{\theta }}^{-1}\left(\frac{1+ \varvec{\alpha }}{2}\right)\right],$$

where N  is minimum sample size, T is number of choice tasks = 6, p is expected choice proportion = 0.5, a is accuracy level = 0.1, α is confidence level = 0.95 and θ^−1^ is the inverse cumulative distribution.

Allowing for an exclusion rate of 8%, based on actual exclusion rates from a previous choice-based survey with this population (24), this equation provides a minimum sample size of 36 respondents per survey version (432 respondents overall). Based on the potential to recruit large numbers of respondents via the MS Register, we aimed to recruit at least 105 respondents per survey version (1260 respondents overall).

### Analysis Methods

Prior to analysis, we identified respondents who provided the same answer to all DCE questions. One of the options in the practice DCE question was dominated by the other (i.e. was ‘worse’ on all attributes); respondents who selected the dominated option were also identified. Two versions of the initial analysis were undertaken, including and excluding these respondents, to ascertain the effects on model performance and coefficients. All analysis was undertaken in Stata 15 (StataCorp LLC, Texas).

Initial analysis was undertaken using a main effects multinomial logit (MNL) model, with categorical attributes coded as dummy variables. The presence of interactions between the Impact attribute and each of the other five attributes was assessed to determine whether changes in the level of one attribute influence the relative importance of another [23]. Interaction terms took a value of 1 where both attributes were at their “worst” level and 0 otherwise. The interaction terms were added to the main effects model, and any that were significant (p < 0.05) were retained, providing an alternative model specification. The Salary attribute was used to calculate the marginal willingness to pay (WTP) across attributes, i.e. how much respondents would be willing to sacrifice from salary in order to gain an improvement in each of the other attributes. Thus, the Salary attribute provided a means to quantify the relative importance of the attributes against a monetary metric [31].

Following the MNL analysis, latent class models (LCMs) were used to explore whether there were groups (classes) of respondents with significantly different preferences across the attributes. Any socio-demographic or clinical variables that had been identified in the results of the systematic literature review as factors that could affect people’s employment choices were considered as potential predictors of class membership. These were age, gender, employment status, job type, MS type and MSIS-29 subscale scores. Due to high rates of missing data for the latter two variables, only gender, age, employment status and job type could be used as predictors of class membership. In order to minimise model complexity, the following variables were recategorised as binary variables: employment status (working or not working) and job type (‘higher managerial, administrative and professional’ or ‘intermediate, routine and manual’).

The selection of model specifications to be considered for the LCMs were informed by the MNL analysis. An appropriate number of classes was determined using the consistent Akaike Information Criterion, CAIC [32] and Bayesian Information Criterion, BIC [33]. Further analysis was undertaken to describe the membership of each class in terms of its sociodemographic characteristics [23]. A preferred specification was selected, based on overall model goodness of fit tests and the sign and significance of attribute coefficients [34]. The CAIC and BIC were used to assess model fit, as these can be directly compared across models with different explanatory variables [35].

## Results

### Descriptive Statistics

In total, 2381 members of the MS Register completed the online survey. Of these, 17 provided the same answer to all DCE questions and 14 answered the practice DCE question incorrectly. Excluding these 31 respondents from the MNL analysis gave better model performance with little effect on the size of model coefficients (reported in Online Resource 2), therefore results reported here are based on the remaining 2350 respondents.

The socio-demographic and clinical characteristics of the sample (Table 1) were broadly representative of PwMS living in the UK in terms of gender, MS type and employment status [7, 27, 28]. When asked to classify their current or most recent job, a large proportion selected the “higher managerial, administrative and professional” (44.30%) or “intermediate” (46.34%) categories. Over 90% of respondents reported finding the DCE questions easy or very easy to understand. 52.17% found it easy or very easy to make choices between Job A and Job B, while 47.45% found this difficult or very difficult.

**Table 1:** Socio-demographic and clinical characteristics of sample

Characteristic	Frequency	Percent
Gender		
Female	1677	71.36
Male	569	24.21
Missing	104	4.43
MS type		
Primary progressive	216	9.19
Relapsing-remitting	1346	57.28
Secondary progressive	386	16.43
Don’t know	156	6.64
Missing	246	10.47
Current employment status		
Employed or self-employed full time	815	34.68
Employed or self-employed part time	547	23.28
Not working for medical reasons	757	32.21
Not working (other)	222	9.45
Missing	9	0.38
Type of job (socio-economic classification)		
Higher managerial, administrative and professional occupations	1041	44.30
Intermediate, routine and manual occupations	1229	52.30
Missing	80	3.40
What were the questions like to understand?		
Very easy	841	35.79
Easy	1307	55.62
Difficult	184	7.83
Very difficult	9	0.38
Missing	9	0.38
How easy or difficult was it to make choices between the jobs?		
Very easy	157	6.68
Easy	1069	45.49
Difficult	1015	43.19
Very difficult	100	4.26
Missing	9	0.38

	Frequency	Mean	SD	Min	Max	Median
MSIS-29 physical	1,771	48.36	19.88	20	100	45
MSIS-29 psychological	1,891	21.08	8.02	9	45	20
Age (years)	2,249	49.30	9.34	22	64	50

The total number of responses differs between characteristics because data was not available for all respondents

Ranges for MSIS-29: physical subscale = 20 to 100; psychological subscale = 9 to 45. On both subscales, lower scores represent better health-related quality of life

### Multinomial Logit Models

Table 2 shows the results of the MNL models that were fitted to the DCE data. In the main effects model, all attributes were significant predictors of respondents’ choices. Further analysis indicated that two interaction terms (Impact * Travel and Impact * Salary) were significant. Adding these to the main effects model slightly improved predictive ability and model fit. The attribute with the greatest effect on respondents’ choices was the impact that working had on other aspects of their lives (coefficient = 1.770). Among the workplace-related attributes, the most important was having understanding and supportive managers and colleagues (coefficient = 1.392). The least important attributes were those relating to physical alterations in the workplace (coefficients = 0.845, 0.493).

**Table 2:** Multinomial logit and latent class models

	Multinomial logit models^a^				Latent class models: three classes				Latent class models: four classes			
	Main effects		With interactions		Main effects		With interactions		Main effects		With interactions	
	Coeff	p-value	Coeff	p-value	Coeff	p-value	Coeff	p-value	Coeff	p-value	Coeff	p-value
Class 1												
Impact	1.770	<0.001	1.787	<0.001	2.085	<0.001	1.568	<0.001	4.415	<0.001	0.882	0.001
Flexibility	1.052	<0.001	1.041	<0.001	1.511	<0.001	1.386	<0.001	−0.188	0.557	0.371	0.058
Culture	1.392	<0.001	1.384	<0.001	3.251	<0.001	3.358	<0.001	4.063	<0.001	2.889	<0.001
Travel	0.907	<0.001	0.998	<0.001	0.825	<0.001	0.649	<0.001	0.049	0.935	0.652	0.001
Alterations level 2	0.493	<0.001	0.475	<0.001	1.045	<0.001	0.996	<0.001	−0.933	0.195	0.600	0.004
Alterations level 1	0.845	<0.001	0.848	<0.001	1.231	<0.001	1.629	<0.001	0.171	0.767	1.110	<0.001
Salary	0.003	<0.001	0.003	<0.001	0.002	0.003	−0.001	0.342	0.000	0.839	0.001	0.401
Impact * Travel			−0.200	<0.001			0.141	0.369			−0.052	0.830
Impact * Salary			0.147	0.008			0.954	<0.001			0.743	0.009
Class 2												
Impact					3.147	<0.001	3.345	<0.001	4.165	<0.001	2.565	<0.001
Flexibility				3.215	<0.001	3.406	<0.001	3.775	<0.001	14.516		
Culture					1.463	<0.001	1.517	<0.001	1.012	<0.001	0.453	0.115
Travel					1.435	<0.001	1.668	<0.001	1.721	<0.001	8.425	<0.001
Alterations level 2					0.576	<0.001	0.655	<0.001	0.414	0.073	−2.261	<0.001
Alterations level 1					1.564	<0.001	1.538	<0.001	1.725	<0.001	1.683	<0.001
Salary					0.005	<0.001	0.007	<0.001	0.007	<0.001	0.017	<0.001
Impact * Travel							−0.402	0.076			1.501	0.002
Impact * Salary							−0.217	0.540			3.251	<0.001
Class 3												
Impact					2.014	<0.001	2.130	<0.001	3.236	<0.001	22.261	<0.001
Flexibility					0.511	<0.001	0.542	<0.001	3.391	<0.001	22.219	<0.001
Culture					0.925	<0.001	0.944	<0.001	4.255		22.791	<0.001
Travel					1.321	<0.001	1.472	<0.001	1.462	<0.001	1.289	<0.001
Alterations level 2					0.403	<0.001	0.359	<0.001	1.444	<0.001	1.262	<0.001
Alterations level 1					0.903	<0.001	0.869	<0.001	1.880	<0.001	1.774	<0.001
Salary					0.005	<0.001	0.004	<0.001	0.003	<0.001	0.002	0.037
Impact * Travel							−0.265	0.005			−0.007	0.971
Impact * Salary							0.228	0.042			0.146	0.569
Class 4												
Impact									1.596	<0.001	2.799	<0.001
Flexibility									0.538	<0.001	0.720	<0.001
Culture									0.937	<0.001	0.844	<0.001
Travel									1.267	<0.001	1.460	<0.001
Alterations level 2									0.433	<0.001	0.357	<0.001
Alterations level 1									0.888	<0.001	0.906	<0.001
Salary									0.005	<0.001	0.004	<0.001
Impact * Travel											− 0.372	<0.001
Impact * Salary											0.102	0.328
Performance												
Observations		28,200		28,200		2168		2168		2168		2168
Respondents		2350		2350		26,016		26,016		26,016		26,016
AIC		12,654.57		12,627.62		11,249.19		11,243.62		11,250.04		11,281.18
BIC		12,712.30		12,701.84		11,218.19		11,206.62		11,207.04		11,230.18

*Coeff* model coefficient

^a^For the multinomial logit model, all respondents are in Class 1

### Latent Class Models

Informed by the MNL analysis, two alternative specifications were considered for the LCMs: a main effects specification and a model including the Impact * Travel and Impact * Salary interaction terms. The CAIC and BIC indicated the presence of three or four distinct classes for each specification (models with between two and eight classes were considered). Therefore, four candidate models were run, with three and four classes for each of the specifications. Table 2 shows that the models with four classes generally had inferior performance according to the BIC and CAIC, and produced some coefficients that were nonsignificant or had unexpected signs. Similarly, some coefficients in the three class model with interaction terms were nonsignificant. Therefore, the preferred model is the main effects model with three classes.

**Fig. 2:**
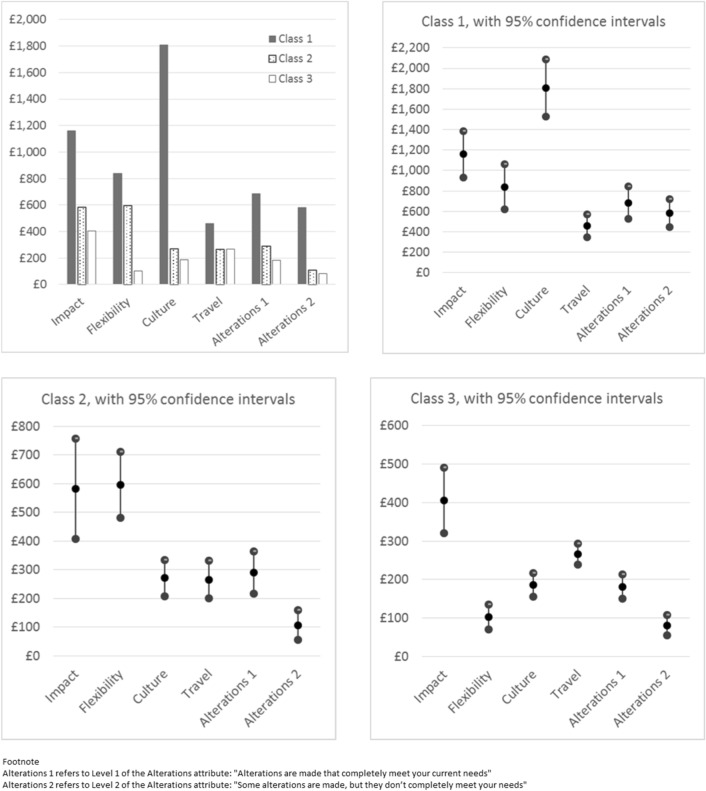
Willingness to pay for attributes by class

Results of marginal WTP and class membership for the preferred model are reported in Table 3 and illustrated in Fig. 2. The three classes differed significantly in terms of age, gender, type of job and type of MS, although the differences in mean age between classes were small (at most 2.5 years). There were no statistically significant differences between the classes in terms of other clinical or sociodemographic variables.

**Table 3:** Main effects latent class model with three classes: willingness to pay and class membership

	Class 1: n=851; 39.25% of sample			Class 2: n=548; 25.28% of sample			Class 3: n=769; 35.47% of sample			Statistics for sociodemographic variables			
Variable	WTP	95% CIs		WTP	95% CIs		WTP	95% CIs					
Impact	£1159.16	£933.04	£1385.27	£583.20	£408.43	£757.98	£405.49	£320.25	£490.73				
Flexibility	£840.19	£621.58	£1058.79	£595.87	£481.40	£710.35	£102.87	£70.43	£135.31				
Culture	£1807.39	£1527.27	£2087.52	£271.10	£207.27	£334.92	£186.16	£155.29	£217.02				
Travel	£458.46	£346.50	£570.42	£265.93	£200.89	£330.96	£265.98	£239.00	£292.95				
Alterations level 2	£581.05	£444.99	£717.11	£106.69	£54.69	£158.70	£81.05	£54.08	£108.01				
Alterations level 1	£684.67	£524.27	£845.08	£289.80	£216.34	£363.26	£181.73	£149.64	£213.82				
Choice probabilities^a^	(0.75)	0.86		(0.71)	0.85		(0.67)	0.70					

Class membership	Number	Percent		Number	Percent		Number	Percent		Total	Total %	Pearson χ^2^	p-value
Gender													
Male	196	23.03		109	19.89		239	31.08		544	25.09	24.4763	<0.001
Female	655	76.97		439	80.11		530	68.92		1624	74.91		
Job type													
NS-SEC 1	362	42.54		274	50.00		357	46.42		993	45.80	7.6621	0.022
NS-SEC 2–3	489	57.46		274	50.00		412	53.58		1175	54.20		
Employment status													
Not working	367	43.13		203	37.04		324	42.13		894	41.24	5.4837	0.064
Working	484	56.87		345	62.96		445	57.87		1274	58.76		
MS type													
RRMS	534	71.49		343	73.76		427	64.02		1304	69.40	14.7969	0.001
Progressive	213	28.51		122	26.24		240	35.98		575	30.60		

Age	Mean	SD		Mean	SD		Mean	SD		Total	SD	F-statistic	p-value
Years	48.19	9.40		48.80	9.45		50.73	8.95		49.24	9.32	16.030	<0.001

*NS-SEC 1* higher managerial, administrative and professional, *NS-SEC 2–3* intermediate, routine and manual

^a^Choice probabilities presented as (unconditional) conditional

851 respondents (39.25%) formed Class 1. Compared to the average across all respondents, members of this class were younger, more likely to be female, to have relapsing-remitting MS, and to classify their most recent occupation as ‘intermediate, routine and manual’. The main determinant of this group’s choices was Culture, followed by Impact. The coefficient for the Salary attribute was lower (0.002) compared to the other two classes (0.005). As Fig. 2 illustrates, this had an important effect when the attribute coefficients were converted into WTP: members of this class were willing to pay more to gain improvements in other attributes.

Class 2 was comprised of 548 respondents (25.28%). Compared to the overall average, these respondents were younger, more likely to be female, to have relapsing-remitting MS, and to classify their most recent job as ‘higher managerial, administrative and professional’. This group’s choices were primarily driven by the Impact and Flexibility attributes.

Compared to the overall average, the 769 respondents (35.47%) who formed Class 3 were older, more likely to be male and to have a progressive form of MS. For this class, Impact had the greatest effect on their choices, followed by Travel.

## Discussion

We report a DCE that explores the relative importance of key factors that may influence whether or not PwMS remain in employment, and how these differ across PwMS. As far as we are aware, this is the first stated preference study to investigate this, expanding our limited understanding of the drivers of workforce participation, an issue with important implications for the wellbeing of PwMS.

All factors included in the DCE were found to be significant predictors of respondents’ choices. Looking at the whole sample, in the MNL model, the attribute with the greatest effect on respondents’ choices was the impact that working had on other aspects of their lives. Other studies have shown that PwMS often need to maintain a delicate balance between the financial and therapeutic advantages of working and possible disadvantages in terms of the impact on their ability to participate in important non-work activities and exacerbations of some MS symptoms [7, 10].

The most important workplace-related attribute for the whole sample was having understanding and supportive managers and colleagues, reflecting the findings of previous studies [7, 9, 10, 13, 15]. According to the APPG report [7], a lack of understanding of MS often results in unhelpful assumptions, which when combined with a lack of effective communication frequently mean that the support provided does not meet the needs of individual employees. The results for the Alterations attribute illustrate the importance of tailoring support to individual needs. Respondents were willing to pay at least an additional £100.68 per month for alterations that completely met their needs (rising to £183.11 for Class 2) compared to alterations that only partially met needs. This highlights the importance of employer attitudes and behaviour in ensuring the appropriateness of job-related accommodations for individual employees with MS.

### Implications of Differences in the Relative Importance of Attributes Between Classes

The LCM analysis revealed considerable variation in preferences across the sample. Considering how the socio-demographic and clinical characteristics of each class of respondents may influence the observed differences in preferences between attributes may provide a useful basis for best practice and policy implications. Key examples of this are discussed below.

While Flexibility (adjustments made to the job to adapt to the employee’s needs) had a greater effect on choices than Alterations (physical adaptations in the workplace) for Class 2, the opposite was true for Class 3 (for Alterations Level 1). Given that members of Class 3 were more likely to be male than those in Class 2, this may reflect the under-representation of women in particular roles and sectors of the economy. The nature of jobs that are ‘traditionally male’ may make physical alterations in the workplace more relevant than flexibility, and vice versa for ‘traditionally female’ jobs. If these differences in preferences are indeed driven by differences in the career choices of men and women, rather than by gender per se, any policy or best practice recommendations should be informed by the nature of the job, rather than the gender of the person performing it.

An alternative explanation is that members of Class 2 were more likely to have relapsing-remitting MS, whereas Class 3 were more likely to have a progressive form of MS. It seems reasonable to assume that the fluctuating nature of relapsing-remitting MS would make flexibility more relevant, enabling employees to self-manage their work in response to changes in their health. For people with secondary progressive, or more established primary progressive MS, the provision of ‘static’ physical aids or adaptations may be more important. It is, however, noteworthy that the proportion of men diagnosed with primary progressive MS exceeds that of women, who are more likely than men to be diagnosed with relapsing-remitting MS [2], making the respective effects of gender and MS type challenging to unravel.

For members of Class 1, the Culture attribute, i.e. having understanding and supportive managers and colleagues, was particularly important. Compared to the overall sample, this group was slightly younger and more likely to describe their occupation as “intermediate, routine or manual”. This may suggest that members of this class were less likely to be in positions of influence, and were therefore more affected (positively or negatively) by the prevailing organisational culture and attitudes. The higher prevalence of relapsing-remitting MS among this group may also play a part. The fluctuating pattern of symptom exacerbation and improvement, alongside ‘invisible’ symptoms such as fatigue [2, 3], may cause challenges for people with relapsing-remitting MS in gaining acceptance and understanding of their needs by managers and colleagues.

Members of Class 1 placed relatively low importance on Salary, producing high marginal WTP for improvements in all other attributes (ranging from £458.46 to £1807.39 per month) when compared to the other two classes and to current UK average monthly earnings of £2210 [36]. Where the total WTP across all attributes for a particular scenario exceed the likely monthly earnings for a job, it is possible that people would not accept a job with this combination of attribute-levels, hence this group may represent those respondents who would have rejected both jobs, if an “opt-out” option had been provided.

### Strengths and Limitations

To our knowledge, this is the largest survey undertaken to date of the employment preferences of PwMS. As such, it provides useful information about how PwMS may be supported to re-enter or remain in the workforce, if they choose to do so. The development of attributes, experimental design and analysis methods were informed by good practice guidance [21, 22, 35], suggesting the DCE was comprehensive in exploring the relative importance of factors. The substantial sample size has enabled us to undertake a detailed exploration of preferences, to produce precise trade-off estimates, and to explore heterogeneity across the sample.

The extent to which the sample represents PwMS on the characteristics of most relevance to the study may be questioned. Notably, a large proportion of respondents described their most recent occupation as “higher managerial, administrative and professional”. This may limit the generalisability of the findings.

The experimental design assumed interactions between most attributes to be zero, which may not be the case. For example, interactions between the alterations variable and job flexibility, or workplace culture, seem reasonable. Resource constraints precluded testing all possible interactions. Nevertheless, the interaction effect that was included in the design had limited impact.

The WTP estimates may be influenced by the choice of levels for the Salary attribute. However, the main point of estimating WTP was to provide information about the relative importance of attributes between the groups, rather than an estimate of how much a person with MS would be willing to pay for an improvement on a particular attribute. It is also possible that the wording of the Salary attribute could be problematic. Respondents were asked about salary in relation to “the average pay for this type of job”. We intended “type of job” to be interpreted in relation to the type of role, sector or industry. However, respondents might have thought about this in terms of the descriptions of the jobs that were provided in the DCE questions. This would be problematic, as they would be taking the levels of the attributes into account when setting their baseline figure for “average pay”. However, other forms of wording that were considered also had potential limitations, and the results indicate that the attribute worked as expected.

The decision to adopt a forced choice design means that respondents were unable to refuse both jobs in any one choice pair (and this may explain the high WTP estimates). Therefore, while the results provide an indication of how a job or working environment might be altered to enable PwMS to remain in the workforce, there is no guarantee that these changes would be sufficient to affect workforce participation. Future studies may wish to include the option to refuse both jobs, possibly for a subset of respondents, in order to explore what proportion would reject both jobs, and whether particular classes of respondents are more or less likely to reject both options.

While the LCM analysis indicates the likely profile of each class in terms of the included characteristics, allocation of respondents across classes is undertaken on a purely probabilistic basis. Therefore, an individual’s classifications on the included characteristics do not mandate their class membership and should not be used to make assumptions about their preferences across the attributes.

### Study Implications

The relative importance of the factors identified here can be used to inform best practice to enable PwMS to stay in employment, if they wish to do so. Overall, the preferences of PwMS for the DCE attributes imply that employers might usefully focus on learning about the potential effects of MS on people’s lives and work, and on developing and maintaining a culture in which PwMS feel supported by management and their peers. However, there appear to be two mediating characteristics that influence which attributes may be more important in a given situation: the nature of the job in question and the type of MS experienced by the employee. This suggests that employers should consider the physical, cognitive and emotional demands of a job role in the context of the symptoms and disease trajectory experienced by the employee in order to ensure that the support offered is relevant. For example, while physically-focused “reasonable adjustments” [37] may be a high priority for job types involving physical demands and people with physical limitations, greater flexibility may be more important for emotionally or cognitively demanding jobs and people with a relapsing-remitting disease course.

These findings support the assertion that a ‘one size fits all’ approach will not deliver meaningful outcomes for PwMS in the workplace. This highlights the importance of ongoing communication between managers and employees, in order to ensure that, where possible, individual needs are recognised and met in a way that suits the employee.

## Conclusions

Employment has been shown to have a positive effect on the wellbeing of people with long-term conditions. This study sheds light on the relative importance of factors that influence the decisions of PwMS with regard to their employment, and the extent to which the importance of these factors varies between PwMS. This can help to inform employers and policy-makers about the steps that may be taken to enable PwMS to remain in the workforce for longer, should they wish to do so.

### Supplementary Information

Below is the link to the electronic supplementary material.

(PDF 358 kb)

(PDF 49 kb)

## Appendix: Introduction to Discrete Choice Experiment (DCE) and Example of a DCE Question

### Introduction to discrete choice experiment

Imagine that you have been looking for a job recently. You have been offered two jobs, Job A and Job B, by two different employers. Both jobs match your skills and experience, and they are identical in all respects, except for the six factors listed below. Please take a moment to read these factors before continuing.

Flexibility of job: This factor relates to adjustments that can be made to the job, to adapt to your needs. These can include working part-time or flexible hours, or changing the amount and type of work you are expected to do.

Alterations in the workplace: This factor relates to alterations that can be made in the workplace. These can include help with getting around at work, making sure you can access facilities (e.g. toilets), and providing specialist equipment.

Workplace culture: This factor relates to attitudes and behaviour in the workplace. This includes how supportive your employer and colleagues are, and how well they understand about how your MS affects you.

Getting to work: This factor relates to your ability to get to and from the workplace. This includes having access to suitable transport and the physical location of the workplace.

Impact of working: This factor relates to the impact of your work on other aspects of your life. This could be positive (e.g. financial security, sense of purpose) or negative (e.g. making some of your symptoms worse, not leaving you enough energy to do other things).

Salary: Take-home pay (after tax deductions).

Please imagine yourself in this situation and make a real decision as to which of the two jobs you would prefer. In making your choice, please read carefully the full description of each job. Please use only the information you are given about the jobs to choose between them. Several aspects of a job may not be mentioned, but it is important that you only use the information used in the job descriptions to make your choice.

Remember: there are no right or wrong answers. We just want to know what you think.

### Example of a DCE question

**Table:**

Imagine that you have been offered two jobs, Job A and Job B. The two jobs are identical in all respects, except for the six factors listed below. Please assume that these are the only two options available to you. Which would you prefer, Job A or Job B?						
	Job A			Job B		
Flexibility of job	The job can be adjusted to accommodate your needs as they change or fluctuate			The job can be adjusted to accommodate your needs as they change or fluctuate		
Alterations in the workplace	Alterations are made that completely meet your current needs			Some alterations are made, but they don’t completely meet your needs		
Workplace culture	Your bosses and colleagues do not understand about your MS and are not supportive			Your bosses and colleagues understand about your MS and are supportive		
Getting to work	It is usually easy to get to the workplace			It is usually easy to get to the workplace		
Impact of working	Overall, work has a positive impact on other aspects of your life			Overall, work has a negative impact on other aspects of your life		
Salary	£100 per month more than the average pay for this type of job			£100 per month less than the average pay for this type of job		
Which job would you prefer?	Job A			Job B		
