# Supplementary material for: Exploring the Factors that Influence Workforce Participation for People with Multiple Sclerosis: A Discrete Choice Experiment
Source: J Occup Rehabil. 2021 Jan 27;31(3):613–26. doi: 10.1007/s10926-020-09952-5 (PMC8298228; doi:10.1007/s10926-020-09952-5)

Exploring the factors that influence workforce participation for people with multiple sclerosis: a discrete choice experiment

Journal of Occupational Rehabilitation

Elizabeth Goodwin, Annie Hawton, Jennifer A. Whitty, Colin Green

Corresponding author: Elizabeth Goodwin, Health Economics Group, Institute of Health Research, University of Exeter, e.goodwin@exeter.ac.uk

Online Resource 1: Development of attributes and levels

This supplementary material provides a brief description of the methods used to develop attributes and levels for a discrete choice experiment (DCE), which aimed to investigate which factors are most important in influencing the employment choices of people with MS. These comprised a systematic review of the literature reporting qualitative research with people with MS that sought to identify the factors that influence their decisions about employment, and a series of workshops undertaken with people with MS to identify the most important factors and to transform these into attributes suitable for a DCE.

Systematic literature review

The aim of the systematic literature review was to identify factors that people with MS have identified as important in influencing their decisions about maintaining, leaving, or making changes to their employment. A systematic search was undertaken to identify papers that described research undertaken to identify such factors. The search terms designed to identify papers about employment were developed from words and phrases used in the abstracts of papers that were located via a scoping search of the Medline database. The search terms for MS were based on those used by the Cochrane Collaboration. A number of databases were searched (Medline, Embase, PsycInfo, CINAHL, ISI Web of Science, ASSIA, EconLit). An example search strategy is provided in Table A1 below.

The inclusion and exclusion criteria for the review were as follows.

Inclusion criteria:

- Qualitative methods
- Original research studies
- People with MS
- Aimed to identify factors that affect whether people with MS are in employment

Exclusion criteria:

- Studies not in English language
- Review articles, comments, editorials or letters
- Published before the year 2000.

Table A1: Search strategy for Medline database

Employment search terms	MS search terms
Profession$.mp.<br>Unemploy$.mp.<br>Vocation$.mp.<br>Job$.mp.<br>Career$.mp.<br>Occupation$.mp. or Occupations/<br>Workplace/<br>Absenteeism/<br>Presenteeism/<br>Presenteeism.mp.<br>Occupational Health/<br>Sick Leave/<br>Occupational Medicine/<br>absenteeism.mp.<br>Productivity.mp.<br>Work/<br>Work Capacity Evaluation/<br>Work Performance/<br>Return to Work/<br>(employed or employer or employment).mp.	exp Multiple Sclerosis/<br>exp Demyelinating Diseases/<br>multiple sclerosis.mp.<br>demyelinating disease*.mp.<br>demyelinating disorder.mp.<br>clinically isolated syndrome.mp.

The search strategy generated 3290 unique records. After screening on the basis of the title and abstract, the full texts of 29 records were assessed against the inclusion and exclusion criteria. Eight papers were included in the review. More detail is provided in Figure A1.

For each study, all the relevant factors that were identified were extracted and listed. The lists for all eight studies were combined into one single list of 38 unique factors. These were then grouped into categories, which were both informed by the themes developed via the analysis reported in the original papers, and generated inductively from analysis of the full list of factors. This process was used to develop the mind-map presented in Figure A2, which formed the basis for the PPI workshops.

PPI Group

A group of six people with MS were recruited from existing PPI groups that advise on research projects at the University of Exeter Medical School and the National Institute for Health Research (NIHR) Collaboration for Leadership in Applied Health Research and Care for the South West Peninsula (PenCLAHRC). The group included people who were currently working full-time or part-time, and people who had permanently left the workforce due to the effects of MS. Group members were balanced in terms of gender, ranged in age from

their 30s to their 60s, and varied in both the severity of their MS and the type of MS with which they were diagnosed (relapsing-remitting, primary progressive or secondary progressive)..

PPI Workshop 1

The purpose of the first PPI workshop was to identify the factors that are most important in influencing the choices of people with MS in regard to their employment. Each participant was provided with their own copy of the mind-map (Figure 1), consisting of the categories and factors printed onto individual cards and arranged on a magnetic whiteboard. Firstly, they were asked to consider whether any of the factors were the same, whether there were any descriptions on the cards that should be split into more than one factor, whether certain factors were related to others, and whether there were any important factors missing. Secondly, participants used their cards and whiteboards to arrange the factors in order of importance to them. After the individual exercises, the participants came back together to produce a joint list agreed by the group and began to organise the selected factors into categories, aiming to generate attributes that would be suitable for a DCE. In total, 23 factors were selected to be categorised into potential DCE attributes.

Following the workshop session, the research team continued the process of categorising the factors, aiming to ensure that the resulting categories met the following criteria (1) suitable for transformation into attributes for a DCE, (2) considered to be of high importance by people with MS and (3) as far as possible, amenable to change by employers or other organisations. The six categories that we produced were emailed to the group members for consultation. In response to the feedback received, a number of amendments were made to the content and descriptions of the categories.

PPI Workshop 2

The purpose of the second PPI workshop was to review the draft attributes, with a greater focus on the three criteria outlined above, and to consider possible levels for each attribute. An initial set of levels for each attribute, drafted by the research team, was provided as a starting point for this part of the workshop. Firstly, participants worked in pairs to consider each of the attributes in turn, including the title of the attribute, its description and the draft levels. We then came back together as a group to discuss our thoughts and to produce a first draft of the attributes and levels.

Following the workshop, the research team reviewed the detailed notes taken during the session, and the draft attributes and levels, with an experienced DCE practitioner. On the basis of this, further amendments were made to the attributes and levels, producing a second draft, which was emailed to the PPI Group for further consultation.

For the PPI group, one of the key factors that they were keen to include in the DCE was the impact of work on other attributes of people's lives. The research team had some concerns about this, as we were concerned that this attribute may be too close to the latent construct that the DCE was aiming to explore. When such an attribute is included in a DCE, it can dominate the results, and the DCE will provide no information about the other attributes. Given the strong opinions that were frequently expressed by the PPI group about the importance of this attribute, we decided to include it but to allow interactions with the other attributes in the experimental design. No problems were apparent with this attribute from the analysis of the pilot data (Coast et al, 2012).

There were other factors (eg specific symptoms of MS) that the PPI Group considered important, but were not suitable for inclusion as attributes in the DCE. These were explored in the form of additional questions to be included at the end of the online survey.

Figure A1: PRISMA diagram for literature review

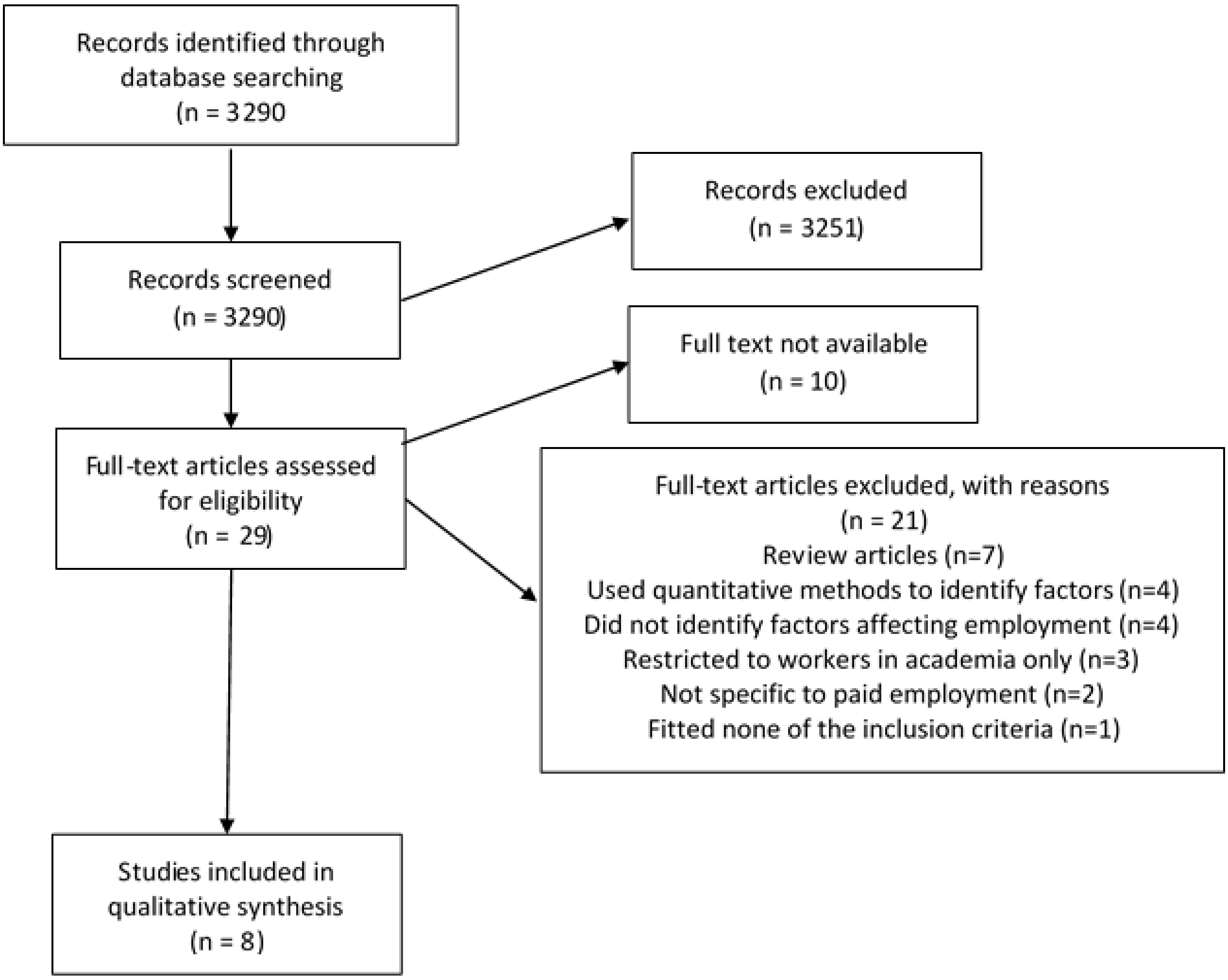

Supplement: Supplementary file 1 — (PDF 358 kb) [file 10926_2020_9952_MOESM1_ESM.pdf]
