# Supplementary material for: Exploring the Factors that Influence Workforce Participation for People with Multiple Sclerosis: A Discrete Choice Experiment
Source: J Occup Rehabil. 2021 Jan 27;31(3):613–26. doi: 10.1007/s10926-020-09952-5 (PMC8298228; doi:10.1007/s10926-020-09952-5)
Supplement: Supplementary file 2 — (PDF 49 kb) [file 10926_2020_9952_MOESM2_ESM.pdf]

Exploring the factors that influence workforce participation for people with multiple sclerosis: a discrete choice experiment. Journal of Occupational Rehabilitation

Elizabeth Goodwin, Annie Hawton, Jennifer A. Whitty, Colin Green

Corresponding author: Elizabeth Goodwin, Health Economics Group, Institute of Health Research, University of Exeter, e.goodwin@exeter.ac.uk

Online Resource 2:
Comparing multinomial logit models with and without excluded respondents

Main effects model, no respondents excluded

Attribute	Coefficient	SE	z	p
Impact	1.728	0.052	32.97	<0.001
Flexibility	1.021	0.026	39.03	<0.001
Culture	1.362	0.028	48.81	<0.001
Travel	0.878	0.027	32.94	<0.001
Alterations 2	0.483	0.030	16.15	<0.001
Alterations 1	0.819	0.036	22.92	<0.001
Salary	0.003	0.000	18.66	<0.001
Respondents	2381			
Observations	28,572			
AIC	12990.72			
BIC	13048.54			
LR $chi^2(7)$	6827.88			
Prob > chi^2	<0.001			
Pseudo R^2	0.345			
Log likelihood	-6488.36			

Main effects model, 31 respondents excluded

Attribute	Coefficient	SE	z	p
Impact	1.770	0.053	33.22	<0.001
Flexibility	1.052	0.027	39.30	<0.001
Culture	1.392	0.029	48.70	<0.001
Travel	0.907	0.027	33.24	<0.001
Alterations 2	0.493	0.030	16.26	<0.001
Alterations 1	0.845	0.036	23.23	<0.001
Salary	0.003	0.000	18.76	<0.001
Respondents	2350			
Observations	28,200			
AIC	12654.57			
BIC	12712.30			
LR $chi^2(7)$	6906.18			
Prob > chi^2	<0.001			
Pseudo R^2	0.353			
Log likelihood	-6320.29			

Model with interactions, no respondents excluded

Attribute	Coefficient	SE	z	p
Impact	1.754	0.065	26.95	<0.001
Flexibility	1.011	0.027	38.12	<0.001
Culture	1.355	0.028	48.45	<0.001
Travel	0.974	0.035	27.94	<0.001
Alterations 2	0.465	0.030	15.45	<0.001
Alterations 1	0.821	0.037	22.47	<0.001
Salary	0.003	0.000	13.22	<0.001
Impact*travel	-0.210	0.046	-4.58	<0.001
Impact*salary	0.141	0.054	2.61	0.009
Respondents	2381			
Observations	28,572			
AIC	12961.46			
BIC	13035.80			
LR $chi^2(9)$	6861.14			
Prob > chi^2	<0.001			
Pseudo R^2	0.346			
Log likelihood	-6471.73			

Model with interactions, 31 respondents excluded

Attribute	Coefficient	SE	z	p
Impact	1.787	0.066	26.94	<0.001
Flexibility	1.041	0.027	38.34	<0.001
Culture	1.384	0.029	48.34	<0.001
Travel	0.998	0.036	28.05	<0.001
Alterations 2	0.475	0.031	15.55	<0.001
Alterations 1	0.848	0.037	22.78	<0.001
Salary	0.003	0.000	13.23	<0.001
Impact*travel	-0.200	0.047	-4.29	<0.001
Impact*salary	0.147	0.055	2.67	0.008
Respondents	2350			
Observations	28,200			
AIC	12627.62			
BIC	12701.84			
LR $chi^2(9)$	6937.13			
Prob > chi^2	<0.001			
Pseudo R^2	0.355			
Log likelihood	-6304.81			

SE = standard error; AIC = Akaike Information Criterion; BIC = Bayesian Information Criterion; LR = likelihood ratio.
